# Aspartic peptidase of *Phialophora verrucosa* as target of HIV peptidase inhibitors: blockage of its enzymatic activity and interference with fungal growth and macrophage interaction

**DOI:** 10.1080/14756366.2020.1724994

**Published:** 2020-02-10

**Authors:** Marcela Q. Granato, Ingrid S. Sousa, Thabatta L. S. A. Rosa, Diego S. Gonçalves, Sergio H. Seabra, Daniela S. Alviano, Maria C. V. Pessolani, André L. S. Santos, Lucimar F. Kneipp

**Affiliations:** a Laboratório de Taxonomia, Bioquímica e Bioprospecção de Fungos (LTBBF), Instituto Oswaldo Cruz (IOC), Fundação Oswaldo Cruz (FIOCRUZ), Rio de Janeiro, Brazil; b Laboratório de Microbiologia Celular, IOC/FIOCRUZ, Rio de Janeiro, Brazil; c Laboratório de Estudos Avançados de Microrganismos Emergentes e Resistentes, Instituto de Microbiologia Paulo de Góes (IMPPG), Universidade Federal do Rio de Janeiro (UFRJ), Rio de Janeiro, Brazil; d Programa de Pós-Graduação em Bioquímica, Instituto de Química, UFRJ, Rio de Janeiro, Brazil; e Laboratório de Tecnologia em Cultura de Células, Centro Universitário Estadual da Zona Oeste (UEZO), Rio de Janeiro, Brazil; f Laboratório de Estrutura de Microrganismos, IMPPG, UFRJ, Rio de Janeiro, Brazil

**Keywords:** Chromoblastomycosis, HIV peptidase inhibitors, aspartic peptidase, antifungal activity, cellular interaction

## Abstract

*Phialophora verrucosa* causes several fungal human diseases, mainly chromoblastomycosis, which is extremely difficult to treat. Several studies have shown that human immunodeficiency virus peptidase inhibitors (HIV-PIs) are attractive candidates for antifungal therapies. This work focused on studying the action of HIV-PIs on peptidase activity secreted by *P. verrucosa* and their effects on fungal proliferation and macrophage interaction. We detected a peptidase activity from *P. verrucosa* able to cleave albumin, sensitive to pepstatin A and HIV-PIs, especially lopinavir, ritonavir and amprenavir, showing for the first time that this fungus secretes aspartic-type peptidase. Furthermore, lopinavir, ritonavir and nelfinavir reduced the fungal growth, causing remarkable ultrastructural alterations. Lopinavir and ritonavir also affected the conidia-macrophage adhesion and macrophage killing. Interestingly, *P. verrucosa* had its growth inhibited by ritonavir combined with either itraconazole or ketoconazole. Collectively, our results support the antifungal action of HIV-PIs and their relevance as a possible alternative therapy for fungal infections.

## 1. Introduction

*Phialophora verrucosa* is a dematiaceous fungus associated with several diseases including chromoblastomycosis (CBM), phaeohyphomycosis and mycetoma^1–3^. However, the main mycosis caused by this fungus is CBM^4^. Although no gold standard therapy for CBM has been proposed, itraconazole is the most commonly used antifungal agent. It also may be combined with other drugs and/or physical methods such as surgery removal and thermotherapy^5^. However, infections caused by CBM fungi, especially *P. verrucosa* are refractory to available therapies and quite difficult to treat^3^^,^^6^. Thus, the main challenges to combat those debilitating fungal infections are the search for new targets and novel therapeutic approaches. Little is known about the mechanisms used by *P. verrucosa* to promote diseases. Most studies are based on taxonomical, clinical and epidemiological researches^6^^,^^7^. Fungal pathogenesis is related to several factors including melanin, dimorphism and hydrolytic enzymes^8^. Enzymes as peptidases are produced by several pathogenic fungi and can modulate essential fungal cell events, such as nutrition, growth, differentiation, biofilm formation, signalling and cell death pathways, as well as invasion and evasion of host cells^9^^,^^10^. In the last years, our research group has shown that *Fonsecaea pedrosoi*, another aetiological agent of CBM, is able to secrete different proteolytic enzymes involved with growth, cell differentiation and fungal pathogenesis^11–15^. In the previous study, we detected an extracellular metallopeptidase activity on *P. verrucosa* and showed that this enzyme could be involved with fungal growth and cellular differentiation^16^.

Direct targeting of peptidases expressed by infectious agents has proven to be a successful therapeutic strategy, notably in the development of hepatitis C virus (HCV) and human immunodeficiency virus (HIV)^17^^,^^18^. Clinical experience has shown the introduction of HIV peptidase inhibitors (PIs) on chemotherapy decreased opportunistic fungal infections mainly caused by *Candida* spp. and *Cryptococcus* spp.^19^^,^^20^. Indeed, several groups have shown that these HIV-PIs are effective in inhibiting the *in vitro* growth of several fungi, including *F. pedrosoi*, *Cryptococcus neoformans Candida albicans* and *Trichosporon asahii*^13^^,^^21–23^. Besides, HIV-PIs were able to affect fungal virulence factors. For instance, indinavir inhibited capsule formation in *C. neoformans*, while amprenavir reduced the biofilm formation in *C. albicans*^21^^,^^22^. Experimental studies showed that HIV-PIs had an effect on those fungi not only *in vitro* but also *in vivo*^9^. Indinavir and ritonavir were able to promote a therapeutic effect in an experimental model of vaginal candidiasis, with an efficacy comparable to the fluconazole treatment^24^. In addition, tipranavir had an inhibitory action in experimental systemic cryptococcosis, reducing fungal burden in the brain and liver of both immunocompetent and immunosuppressed mice^25^. Taking into consideration all the beneficial effects of HIV-PIs, herein, we aimed to investigate the secretion of aspartic peptidase from *P. verrucosa* cells as well as to evaluate the effects of HIV-PIs on its enzymatic activity. In parallel, fungal growth and the interaction of conidial cells with human macrophages were assayed in the presence of the HIV-PIs in order to evaluate their implication to block both relevant biological processes.

## 2. Materials and methods

### 2.1. Fungal growth conditions

*P. verrucosa* isolated from a human patient with CBM^26^ was maintained in Sabouraud dextrose agar (SDA) medium with mineral oil at 4 °C. For all assays, fungal cells were cultivated for 7 days under constant agitation (130 rpm) at 26 °C in 100 mL of yeast nitrogen base (YNB) medium supplemented with 5% dextrose. Conidia were collected using gauze filtering and centrifuged at 4,000 ×*g* for 10 min. The fungal cells were then washed three times with saline (0.85% NaCl) and the number of conidia was estimated using a Neubauer chamber^26^.

### 2.2. Extracellular proteolytic activity detection

The fungal culture (100 mL) was centrifuged, the supernatant filtered through a 0.45 µm membrane (Millipore, MA, USA), and peptidase activity detected as described by Palmeira et al^13^. The cell-free culture supernatant was concentrated 100-fold in a 10,000 molecular weight cut-off Amicon micropartition system (Beverly, MA, USA). For enzymatic class identification, 15 µL of concentrated supernatant (1 µg of protein) and 1.5 µL of human serum albumin (HSA, 1 mg/mL) were incubated for 20 h at 37° C in 20 mM sodium acetate buffer, pH 3.0, supplemented with different proteolytic inhibitors: pepstatin A (10 µM), 1,10-phenanthroline (10 mM), L-*trans*-epoxisuccinil leucilamido-(4-guanidino)butane (E-64, 10 µM) and phenylmethylsulfonyl fluoride (PMSF, 10 mM). Then, 15 µL of sodium dodecyl sulfate-polyacrylamide gel electrophoresis (SDS-PAGE) sample buffer (125 mM Tris, pH 6.8, 4% SDS, 20% glycerol, 0.002% bromophenol blue and 10% β-mercaptoethanol) were added to the reaction mixtures and boiled at 100 °C for 5 min. The control system was prepared with the culture supernatant incubated at the same conditions but without inhibitors. Next, all the reaction systems were subjected to SDS-PAGE. This assay was carried out at 4 °C, 120 V for 1.5 h. The degradation protein profiles were detected by silver staining as described by Blum^27^. Densitometric quantification of the polypeptide bands was performed using the free ImageJ software. Sample normalisation was performed using protein dosage^28^. All PIs were obtained from Sigma-Aldrich Chemical Co (St Louis, MO, USA) and dissolved in dimethyl sulfoxide (DMSO), except pepstatin A that was dissolved in methanol.

### 2.3. Effect of aspartic PIs on Phialophora verrucosa enzymatic activity

Peptidase activity was determined using 7-methoxycoumarin-4-acetyl (MCA)-Gly-Lys-Pro-Ile-Leu-Phe-Phe-Arg-Leu-Lys(DNP)-D-Arg-amide (cathepsin D fluorogenic substrate, Sigma-Aldrich Chemical Co) as described by Santos et al.^29^. The assay was performed in triplicate using a 96-well microtiter plate. Briefly, the reaction was started by the addition of substrate (12 µM) to fungal concentrated supernatant (1 µg of protein) in a buffer containing 100 mM sodium acetate, pH 4.7, 1 M sodium chloride, 1 mM ethylenediamine tetraacetic acid (EDTA), 1 mM dithiothreitol (DTT), 10% DMSO and 1 mg/mL bovine serum albumin (BSA). The system was treated with pepstatin A (10 µM) or 100 µM of HIV-PIs (amprenavir, atazanavir, indinavir, lopinavir, nelfinavir, ritonavir or saquinavir) and a non-treated system was used as control. After 30 min, the cleavage of the cathepsin D substrate was detected in a spectrofluorimeter (FlexStation 3, Molecular Devices, CA, USA) with 328 nm excitation and 393 nm emission wavelengths. The proteolytic activity was calculated based on a standard curve of MCA fluorophore. Protein concentration was measured using the method described by Lowry et al.^28^. All HIV-PIs were purchased from National Institutes of Health (NIH, MA, USA) and dissolved in DMSO. All buffer reagents were obtained from Sigma-Aldrich Chemical Co (St Louis, MO, USA).

### 2.4. Effect of HIV-PIs on Phialophora verrucosa growth

*Phialophora verrucosa* conidia (5 × 10^2^ cells) were incubated with 400 µM of amprenavir, atazanavir, indinavir, saquinavir, lopinavir, nelfinavir and ritonavir. The last three inhibitors were also tested at lower concentrations (200 and 100 µM). In addition, conidia were treated with 50 µM of ritonavir. After 20 h at 26 °C, 100 µL (10^2^ conidia) of each system were plated onto YNB medium supplemented with 2% agar and incubated for 6 days at 26 °C. Fungal growth was estimated using colony-forming units (CFU) quantification^13^.

### 2.5. Effect of HIV-PIs on Phialophora verrucosa ultrastructure

Conidia (1 × 10^6^ cells) were incubated for 20 h at 26 °C in Roswell Park Memorial Institute (RPMI, Invitrogen, Camarillo, CA, USA) 1640 medium in the absence (control) or presence of lopinavir (400 µM), nelfinavir (400 µM) and ritonavir (200 and 400 µM). Subsequently, the fungal cells were processed by scanning electron microscopy (SEM). Briefly, conidia were fixed with 4% paraformaldehyde and 2.5% glutaraldehyde in 0.15 M sodium cacodylate buffer, pH 7.2, at 26 °C for 2 h. Then, cells were post-fixed for 1 h at 26 °C with 1% osmium tetroxide and dehydrated through an ascending series of ethanol ending in 100%. Finally, conidia were dried using a critical point method, mounted on stubs, coated with gold and observed using a Jeol JSM 6490LV scanning electron microscope^26^.

### 2.6. Effect of HIV-PIs on Phialophora verrucosa-macrophage interaction

#### 2.6.1. Culturing animal cells

Human monocytic leukaemia THP-1 cell line (ATCC TIB-202) was cultured in RPMI 1640 medium supplemented with 10% foetal bovine serum (FBS) at 37 °C with 5% CO_2_. Animal cells (4 × 10^5^/mL) were plated in a 24-well cell culture plate containing the same medium added with phorbol myristate acetate (PMA, 80 nM) for monocyte differentiation into macrophages. After 24 h, a new RPMI medium was replaced and the cells were incubated for additional 24 h before the interaction assay^30^.

#### 2.6.2. Cytotoxicity assay

The effect of HIV-PIs on the viability of macrophages derived from THP-1 was determined using 3–(4,5-dimethythiazol-2-yl)-2,5-diphenyl tetrazolium bromide (MTT, Sigma-Aldrich Chemical Co) reduction assay^31^. Briefly, macrophages (4 × 10^5^ cells/mL) were incubated for 20 h in a 96-well culture plate with lopinavir or ritonavir, both at concentrations 25, 50, 100 and 200 µM. Alternatively, the macrophages were treated with two different combinations of HIV-PIs: 100 µM lopinavir plus 25 µM ritonavir and 50 µM lopinavir plus 12.5 µM ritonavir. After that, the macrophages were washed and incubated with 0.5 mg/mL of MTT for 3 h at 37 °C. Then, formazan salt formed was dissolved in DMSO and measured spectrophotometrically at 490 nm. Macrophages incubated at the same conditions but without inhibitors were used as controls.

#### 2.6.3. Fungi-host cell interaction

The conditions performed in this set of experiments were similar to those previously published by Palmeira et al.^13^. Simultaneously, two 24-well culture plates were prepared to determine the adhesion index and macrophage killing. Briefly, viable fungal cells were washed in RPMI and incubated with macrophages at a ratio of 5:1 (fungi:macrophage) for 1 h at 37 °C. Non-associated fungi were then removed and the systems were washed in RPMI medium. Next, the interaction systems were incubated in RPMI medium for additional 20 h with lopinavir (100 µM), ritonavir (25 µM) or a combination of lopinavir (50 µM) plus ritonavir (12.5 µM). The control systems were performed at the same conditions but without inhibitors. After incubation, all systems were washed three times in PBS to remove non-adherent conidia. One cell culture plate was fixed in Bouin’s solution and stained with Giemsa. The infected cells percentage was defined after counting 200 cells per coverslip. Then, the adhesion index was calculated by multiplying the mean number of attached fungi per macrophages by the percentage of infected macrophages. The images of the interaction between *P. verrucosa* conidia and macrophages were obtained using an Olympus BX40F4 microscope. The other cell culture plate was added with sterile cold water to lyse the macrophages and then the suspensions were plated onto SDA medium in order to count the number of CFU (killing assay)^13^.

### 2.7. Effect of ritonavir combined with antifungal drugs on Phialophora verrucosa development

*P. verrucosa* conidia (5 × 10^2^ cells) were incubated with ritonavir (50 µM), individually or in combination with antifungal agents such as amphotericin B (2.5 µM), ketoconazole (5 µM), itraconazole (1.25 µM) and terbinafine (5 µM), which were used at subinhibitory concentrations. After treatment for 20 h at 26 °C, 100 µL (10^2^ conidia) of each system were plated onto YNB medium supplemented with 2% agar for counting the CFU^13^. All antifungal drugs were obtained from Sigma-Aldrich Chemical Co (St Louis, MO, USA) and dissolved in DMSO.

### 2.8. Statistical analysis

All experiments were performed in triplicate in three independent experimental sets. The graphics and data were constructed and analysed statistically by means of Student’s *t*-test using GraphPad Prism 5.01 software. *p* values of 0.05 or less were assumed as significant.

## 3. Results

### 3.1. Phialophora verrucosa conidia secrete aspartic-type peptidase

**Figure 1.**
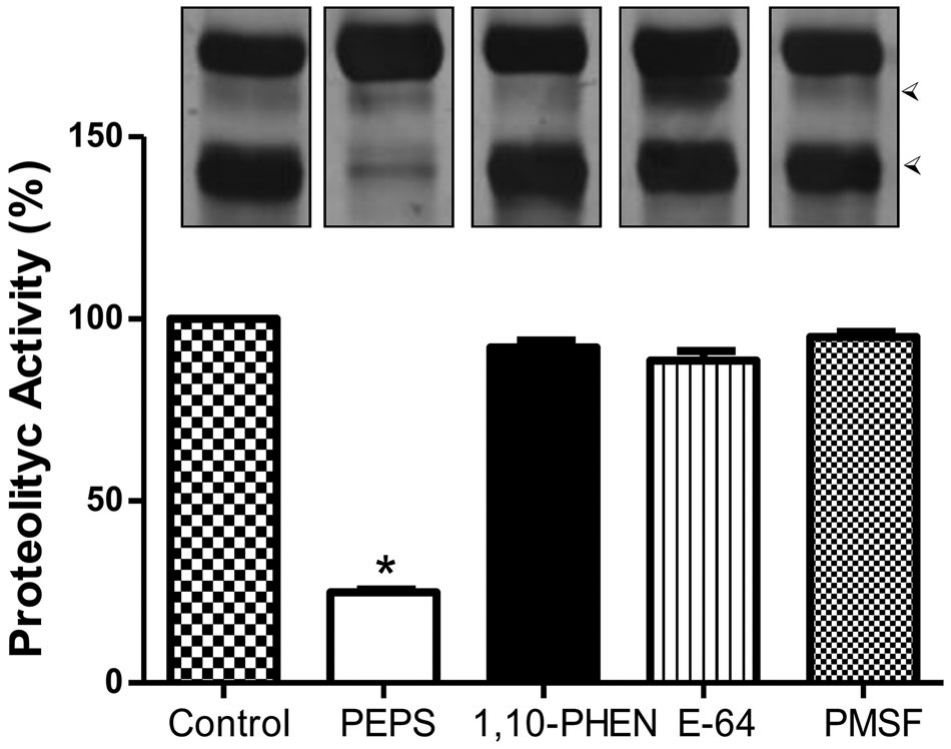
Aspartic peptidase activity in Phialophora *verrucosa* conidia. Concentrated supernatant and HSA substrate were incubated with pepstatin A (PEPS, 10 µM), 1,10-phenanthroline (1,10-PHEN, 10 mM), E-64 (10 µM), PMSF (10 mM) and without inhibitors (control). The degradation profile was analysed using SDS-PAGE. The substrate degradation after hydrolysis is indicated by the arrowheads on the right. Bar graph represents the densitometric analysis of the gel bands using ImageJ software. Asterisks indicate *p* values ≤ 0.05 in comparison with the control system.

Our results revealed that supernatant from *P. verrucosa* conidial cells grown in YNB medium was able to hydrolyse HSA at acidic pH as judged by the SDS-PAGE assay (Figure 1, control). The substrate degradation was not affected by 1,10-phenanthroline, E-64 and PMSF, which are classical inhibitors of metallo-, cysteine- and serine-type peptidases, respectively (Figure 1). Conversely, the enzymatic activity was strongly inhibited by pepstatin A, indicating for the first time that this fungus secretes an aspartic-type peptidase (Figure 1). Corroborating these data, *P. verrucosa* secretion contained a peptidase able to hydrolyse a specific aspartic peptidase substrate, cathepsin D, releasing about 38,000 µM of MCA per mg protein after 30 min of reaction, and pepstatin A inhibited it by approximately 90%, supporting the specificity of the enzymatic reaction (Table 1).

**Table 1.** Effect of HIV-PIs on the peptidase activity secreted by *Phialophora verrucosa*.

Inhibitor	% Relative activity
None	100.00 ± 0.50
Pepstatin A	12.65 ± 3.35
Amprenavir	23.27 ± 9.82
Atazanavir	54.00 ± 1.00
Indinavir	48.50 ± 4.50
Lopinavir	24.15 ± 0.73
Nelfinavir	63.40 ± 10.40
Ritonavir	24.10 ± 7.90
Saquinavir	58.75 ± 6.25

The proteolytic activity was detected using cathepsin D fluorogenic substrate as described in Material and Methods. Proteolytic activity was expressed considering the control value (38,000 μM methylcoumarin/mg/30 min) that was taken as 100%. The proteolytic activity measured in the presence of pepstatin A (10 µM) and HIV-PIs (100 µM) showed hydrolysis significantly different from control (*p* < 0.05, Student’s *t* test), except for nelfinavir.

### 3.2. HIV-PIs inhibited the Phialophora verrucosa peptidase activity, growth and ultrastructure

Lopinavir, ritonavir and amprenavir were the most effective HIV-PIs, inhibiting the aspartic-type peptidase activity released by *P. verrucosa* conidia at about 75% (Table 1), while atazanavir, indinavir and saquinavir inhibited the enzymatic activity around 40–50%. However, this peptidase activity was not significantly inhibited by nelfinavir under the experimental conditions employed herein (Table 1).

**Figure 2.**
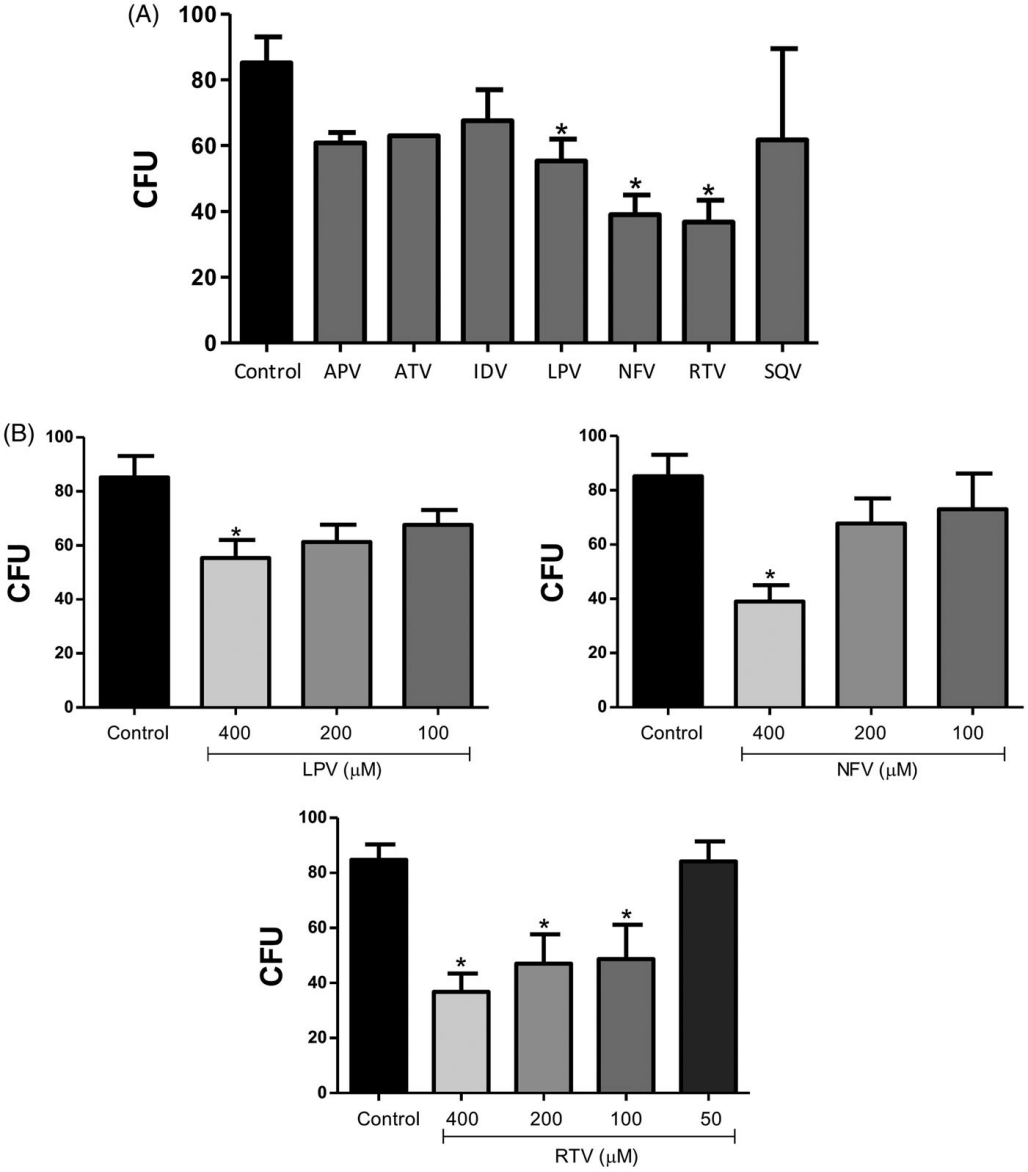
Effect of HIV-PIs on *Phialophora verrucosa* growth. Conidia were incubated for 20 h (A): with 400 µM of amprenavir (APV), atazanavir (ATV), indinavir (IDV), lopinavir (LPV), nelfinavir (NFV), ritonavir (RTV), saquinavir (SQV), or in the absence of inhibitors (control) and (B): with variable concentrations of LPV, NFV or RTV. Growth inhibition in all the systems was determined using the colony-forming unit (CFU) assay. The values represent the mean standard deviation of the three independent experiments performed in triplicate. Asterisks indicate *p* values ≤ 0.05 in comparison with the control system.

The potential antifungal activity of HIV-PIs (400 µM) was assessed and among the HIV-PIs tested only lopinavir, nelfinavir and ritonavir were able to significantly reduce the fungal growth by around 40, 55 and 60%, respectively (Figure 2(A)). The action of these PIs at different concentrations was also tested. Ritonavir was the only one capable of promoting significant fungal growth inhibition in a typically dose-dependent manner, with a decrease of 60, 45 and 40% at 400, 200 and 100 µM, respectively (Figure 2(B)), displaying a half-maximal inhibitory concentration (IC_50_) of 141.42 µM.

**Figure 3.**
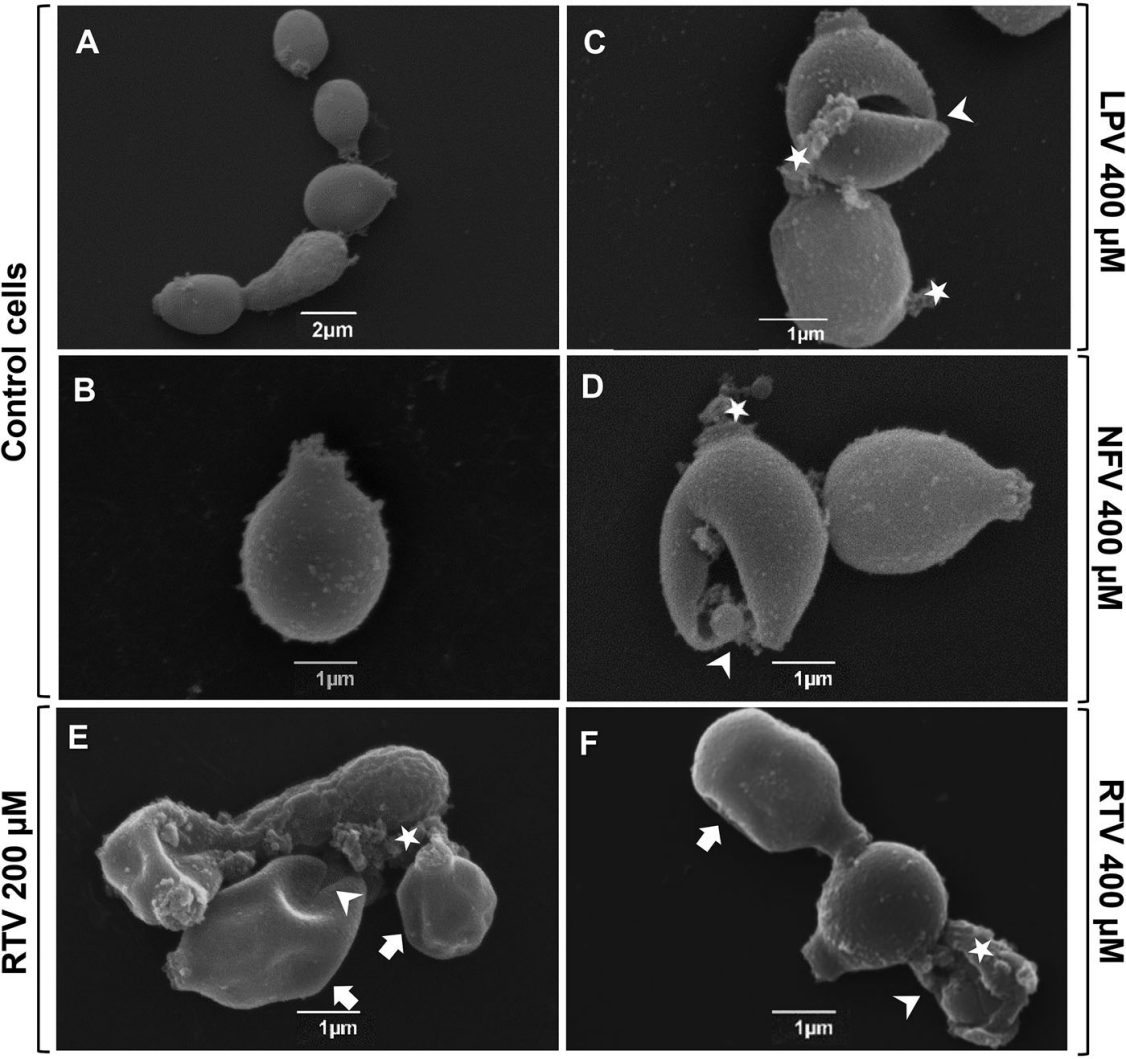
Effect of HIV-PIs on *Phialophora verrucosa* ultrastructure. Conidia (1 × 10^6^) were incubated for 20 h in RPMI medium in the absence (control) or presence of HIV-PIs and processed by scanning electron microscopy (SEM) as described in Material and Methods. Representative images show untreated (control cells, A, B) and treated cells with lopinavir 400 µM (C); nelfinavir 400 µM (D) ritonavir 200 µM and 400 µM (E, F). SEM analyses demonstrated that HIV-PIs treatment induced different cellular alterations, such as cell re-entrances (E, F; arrows), cell disruption (C-F; arrowheads) and surface deposits (C–F; star). Images were obtained using JEOL JSM-6490 LV scanning electron microscope.

The effective action of HIV-PIs was corroborated by SEM (Figure 3) that revealed irreversible ultrastructural alterations when compared with untreated cells, which had typical spherical-to-oval morphology (Figure 3(A,B)). Cells treated with HIV-PIs exhibited several morphological changes, such as surface invagination and surface containing deposits and cell disruption (Figure 3(C–F)), which are indicative of cell death.

### 3.3. HIV-PIs affected Phialophora verrucosa-macrophage interaction

**Figure 4.**
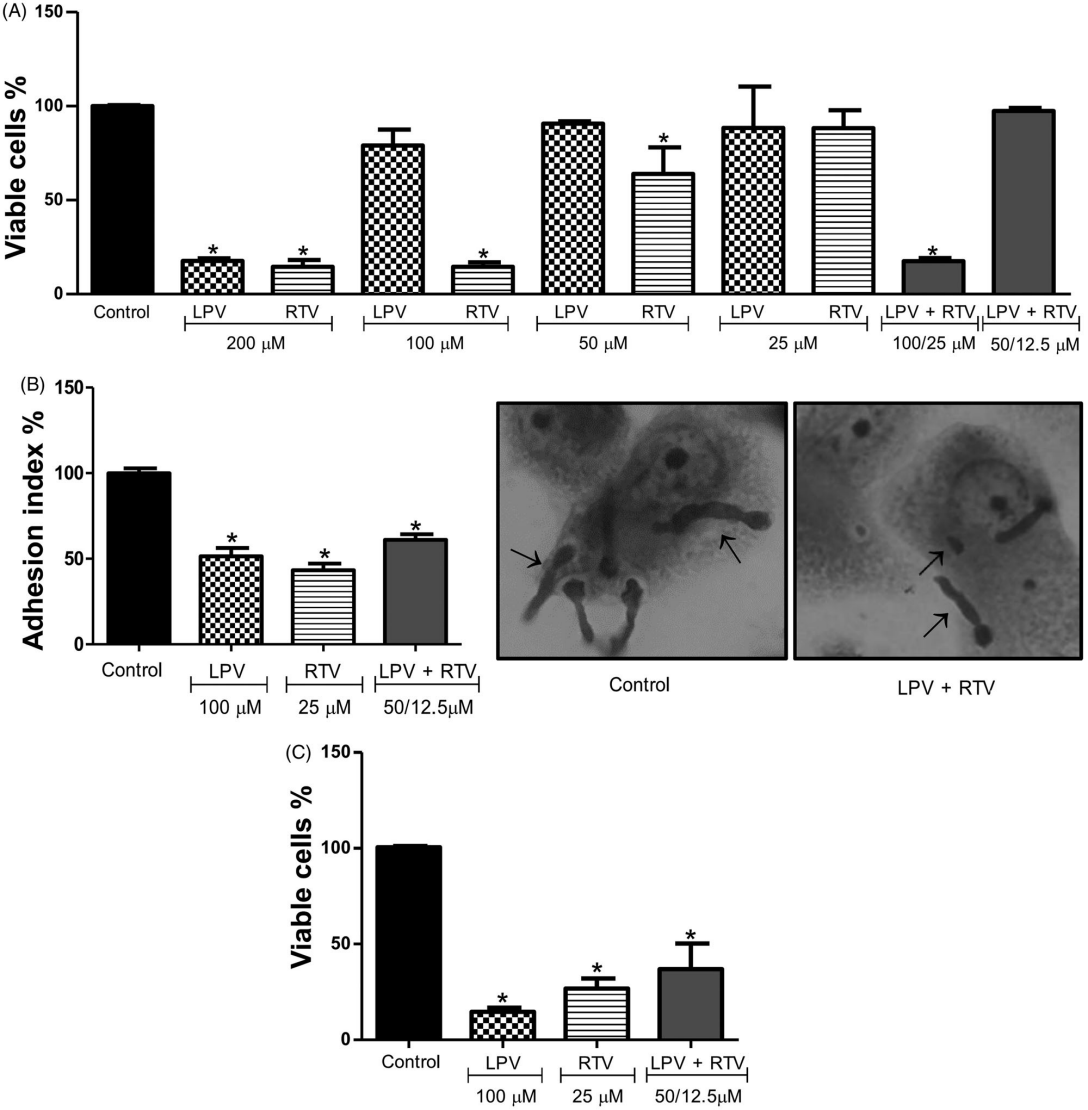
Effect of HIV-PIs on THP-1 viability and *Phialophora verrucosa*-macrophages interaction. (A) THP-1 macrophages (4 × 10^5^/mL) were incubated with lopinavir (LPV) and ritonavir (RTV), individually or in combination, at different concentrations for 24 h and in the absence of inhibitors (control). After treatment, macrophage viability was determined using the MTT assay. Alternatively, THP-1 cells were infected with *P. verrucosa* conidia at a ratio of 5:1 (fungi:macrophage) for 1 h and then non-adherent fungi were removed. The cultures were incubated for additional 20 h with non-cytotoxic concentrations of lopinavir and ritonavir, individually or in combination, and in the absence of inhibitors (control). (B) Adhesion index and (C) macrophage killing. The results were expressed considering the viability of control (untreated cells) as 100%. Asterisks indicate *p* values ≤0.05 in comparison with the control system. Inset: Representative images of attached fungus (arrows) to macrophages are shown by light microscopy analyses.

Our data revealed that only lopinavir at concentrations >100 µM and ritonavir at concentrations >25 µM had a significant deleterious effect on THP-1 viability (Figure 4(A)). When lopinavir (100 µM) and ritonavir (25 µM) were combined, the macrophage viability was affected. However, half concentration of both PIs kept the phagocytes’ viability preserved (Figure 4(A)). Giemsa-stained assay showed that all HIV-PIs were able to disturb the adhesion between *P. verrucosa* conidia and macrophage cells (Figure 4(B)). We observed that ritonavir (25 µM) was the most potent HIV-PI in inhibiting the adhesion event by approximately 60%. Lopinavir at 100 µM also diminished the interaction process at about 50%. In addition, the combination of lopinavir (50 µM) plus ritonavir (12.5 µM) affected the adhesion index by approximately 40% (Figure 4(B)). Furthermore, the killing capability of macrophages against *P. verrucosa* after treatment with HIV-PIs was investigated. In this assay, *P. verrucosa* conidia associated to macrophages were treated with non-cytotoxic concentrations of lopinavir and ritonavir, individually and in combination. Conidia treated with lopinavir and ritonavir, in both conditions, were more susceptible to macrophages than non-treated conidia (control). When lopinavir (100 µM) and ritonavir (25 µM) were incubated individually, they reduced the intracellular conidial viability about 85% and 70%, respectively (Figure 4(C)). In combination, these HIV-PIs were able to reduce about 60% the *P. verrucosa* viability even at subinhibitory concentrations (Figure 4(C)).

### 3.4. Ritonavir combined with antifungal agents inhibited the Phialophora verrucosa proliferation

**Figure 5.**
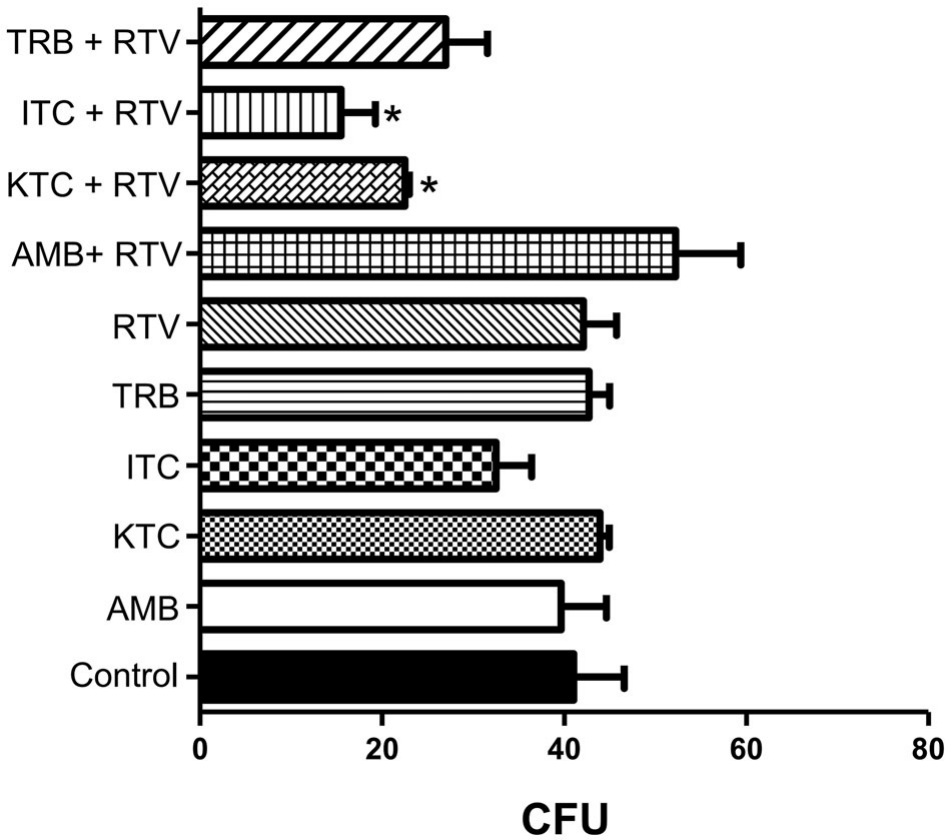
Effect of ritonavir combined with antifungal drugs on *Phialophora verrucosa* growth. Conidia (5 × 10^2^) were incubated with ritonavir (RTV, 50 µM), individually or in combination with amphotericin B (AMB, 2.5 µM), ketoconazole (KTC, 5.0 µM), itraconazole (ITC, 1.2 µM) or terbinafine (TRB, 5.0 µM), and in the absence of drugs (control) at 26 °C for 20 h at subinhibitory concentrations. All antifungal drugs were also tested individually at the same concentrations. Then, the growth inhibition was determined using colony-forming unit (CFU) assay. The values represent the mean standard deviation of the three independent experiments performed in triplicate. (*p < 0.05; Student *t* test).

The association of ritonavir with different classical antifungal drugs, such as ketoconazole, itraconazole, amphotericin B and terbinafine, was also evaluated. As expected, all of them at subinhibitory concentrations did not inhibit the fungal growth when they were tested individually (Figure 5). However, when ritonavir was associated with either ketoconazole or itraconazole, also at subinhibitory concentrations, *P. verrucosa* proliferation was inhibited by approximately 40% and 60%, respectively (Figure 5). These results suggest a possible beneficial combinatory effect between ritonavir and both antifungal drugs. Nonetheless, associations between ritonavir and amphotericin B or terbinafine did not alter the fungal proliferation (Figure 5).

## 4. Discussion

Diseases caused by black fungi, including those responsible for CBM, have promoted several cases of morbidity worldwide^31^^,^^32^. In the last years, our group had studied different structures associated with CBM fungal pathogenesis^11–16^^,^^33–36^. Among them, we described extracellular peptidase activities in *F. pedrosoi* and showed their involvement with growth, differentiation and cellular interaction^13^^,^^14^^,^^36^. *F. pedrosoi* conidia grown in Czapek-Dox secreted mainly aspartic-type peptidase, while *P. verrucosa* conidia secreted metallopeptidase^13^^,^^16^. In this current work, we tested several culture media (data not shown) and the aspartic peptidase activity was detected in concentrated supernatant of *P. verrucosa* conidia from YNB medium. This same minimal medium was also used to induce aspartic peptidase activity of *C. neoformans* required for its survival and virulence in acidic environments^37^. It is well known that the composition of the nutrient medium can modulate the synthesis of different bioactive molecules. Corroborating this statement, *F. pedrosoi* conidial cells are able to secrete two distinct classes of extracellular peptidases: aspartic peptidase when grown in chemically defined medium and metallopeptidase when cultivated in Kauffman complex medium^11^. These results corroborate that the medium composition could modulate the synthesis and secretion of proteolytic enzymes as previously observed for other pathogenic fungi including *C. neoformans*, *C. albicans*, *Scedosporium apiospermum* (formerly *Pseudallescheria boydii*) and *Aspergillus fumigatus*^37–40^. Previous studies reported that peptidase activities can change in response to environmental conditions, which are beneficial for microbial cells adaptation, including inside the host^36^^,^^37^.

Peptidases have emerged as potential targets to the development of new antifungal chemotherapeutics^9^. Several studies have shown that fungal aspartic peptidases are also sensitive to inhibitors produced against HIV used in highly active antiretroviral therapy^13^^,^^41^^,^^42^. Thus, the ability of these PIs to modulate essential events in fungal cells has been investigated^9^^,^^23^. In this work, we revealed that HIV-PIs affected *P. verrucosa* aspartic peptidase activity as we previously demonstrated in *F. pedrosoi*. Among the HIV-PIs tested, saquinavir, nelfinavir and ritonavir were the most effective in inhibiting aspartic peptidase activity of *F. pedrosoi* conidial, mycelial and sclerotic cells^11–14^. Although *P. verrucosa* aspartic peptidase activity has been affected by all HIV-PIs tested, it was not significantly inhibited by nelfinavir. These results showed that aspartic peptidases released by *F. pedrosoi* and *P. verrucosa* had distinct sensibilities to HIV-PIs, suggesting differences between biochemical properties of these enzymes. The action of HIV-PIs on proteolytic activities of other pathogenic fungi such as *C. albicans*, non-*albicans Candida* species, *C. neoformans* and *Trichosporon* spp. was also described^9^^,^^23^^,^^43^^,^^44^.

Several groups have reported that HIV-PIs showed antifungal effects *in vitro* and *in vivo*^9^^,^^21^^,^^25^^,^^45^. Moreover, our group have already demonstrated the antifungal activity of HIV-PIs against *F. pedrosoi*^12–15^. In the present study, we revealed that HIV-PIs were also able to inhibit *P. verrucosa* growth. Lopinavir, nefinavir and ritonavir inhibited *P. verrucosa* proliferation at maximum concentration (400 µM) tested. However, ritonavir was the most potent inhibiting the activity even at 100 µM. Studies conducted by different group have revealed the effective action of HIV-PIs on fungal growth, suggesting that aspartic peptidase can be target of these inhibitors^9^^,^^23^^,^^38^. Thus, the blockage of aspartic peptidases may result in the cells inability to obtain peptides and amino acids for nutrition, affecting directly their development^9^. It is important to emphasise that HIV-PIs may be responsible for multifactorial effects that disturbing fungal homeostasis and culminates in cell death^9^^,^^14^^,^^22^.

There are very few studies regarding HIV-PIs structure-fungal aspartic peptidase activity relationship. Theoretical studies with *Candida* secreted aspartic peptidases (Saps) have suggested that ritonavir interact with the catalytic residues Asp_32_/Asp_218_ of *C. albicans* Sap2 and Asp_32_/Asp_220_ (as Asp_32_/Asp_218_ in Sap2) of *Candida parapsilosis* Sapp1 showing that these interactions are similar to that between ritonavir and HIV-1 aspartic peptidase^9^^,^^46^. Although the interactions are preserved, even slight alterations as electrostatic and hydrophobic differences, in the enzyme active site can affect the binding and the inhibitory efficiency^9^. FDA-approved HIV-PIs were designed to viral peptidase and because of this have a lower affinity for fungal aspartic peptidase. In fact, the inhibitory effect of HIV-PIs against fungal aspartic peptidase is commonly reached in the range of micromolar, like observed to *P. verrucosa* (IC_50_ 141.42 µM) and other fungi, instead of nanomolar as required for HIV aspartic peptidase inhibition^47^. Then, these aspartic peptidases have differences from catalytic properties, biological functions, cellular localisation and inhibition profile^9^. Thus, inhibitory potential may be due to the specificity to fungal aspartic peptidases, and/or the ability to block all members of the Sap family, or at least those Saps most important for virulence, for instance^48^.

In addition, our results also revealed that HIV-PIs had different inhibition profiles from CBM aetiological agents. For instance, ritonavir (100 µM) was more effective in inhibiting *P. verrucosa* growth than *F. pedrosoi*. This fungus proliferation was inhibited by around 90% after treatment with 100 µM of saquinavir and nelfinavir^13^, while they did not affect *P. verrucosa* growth at this concentration. The results obtained using SEM revealed drastic changes on the *P. verrucosa* morphology after HIV-PIs treatment, corroborating their anti-proliferative properties. Our previous study using transmission electron microscopy showed that HIV-PIs also caused irreversible ultrastructure alterations on *F. pedrosoi*^14^. In fact, conidia exposed especially to saquinavir and nelfinavir had morphological changes, such as amorphous material from the cell wall, numerous undulations and/or invaginations on the membrane as well as withdrawal of the cytoplasmic membrane^14^. Likewise, studies using SEM revealed drastic changes on the morphology of *C. albicans* yeasts treated with amprenavir. These analyses demonstrated that treatment with this HIV-PI induced alterations in the cells shape, including invaginations and detachment of the external fibril layer^22^^,^^42^.

The microbial virulence is directly associated to the organism ability to survive and multiply intracellularly^49^. Previous studies showed that CBM fungi, including *P. verrucosa* are able to survive and proliferate inside macrophages^50^^,^^51^. Bearing this in mind, we investigated the adhesion between *P. verrucosa* conidia and THP-1 cells and the killing of macrophages after HIV-PIs treatment. Our data showed that treatment with non-cytotoxic concentrations of lopinavir and ritonavir, individually or in combination, was effective in reducing conidia adhesion to macrophages as well as fungal growth. Our group previously demonstrated that HIV-PIs, such as ritonavir, nelfinavir, indinavir and saquinavir were able to diminish the adhesion and invasion capabilities of *F. pedrosoi* conidia during the interaction with fibroblasts and murine macrophages cell line RAW 264.7^13^. Moreover, the increasing of conidia susceptibility after interaction with murine macrophage was also observed after treatment of *F. pedrosoi* with ritonavir, nelfinavir and indinavir^13^. Castilho et al.^30^ showed that pepstatin A modulated the interaction between *Paracoccidioides brasiliensis* and THP-1 macrophages. As observed in *P. verrucosa* assay with HIV-PIs, yeasts of *P. brasiliensis* treated with pepstatin A were more susceptible to human macrophages than untreated cells. These findings suggest the involvement of proteolytic enzymes that are important virulence factors in fungal cells^9^. It can now be explained, at least in part, due to the significant modulation of important surface molecules, including aspartic peptidase, melanin and glucosylceramide^14^. Thus, aspartic peptidase might be disrupting host cells defence mechanisms affecting the integrity of their important proteins, and other essential physiological processes for pathogen survival. The susceptibility increase of HIV-PIs-treated *P. verrucosa* to macrophages could also be related with important mechanisms that mediate the antimicrobial immunity of phagocytic cells, including the enhancement of oxidative burst as reported to *C. albicans*^52^. It is important to highlight the viable number of *P. verrucosa* conidia associated to macrophages also decreased after treatment with lopinavir and ritonavir together. In clinical practice, for instance, HIV-PIs are administered in combination with antiretroviral regimens for acquired immunodeficiency syndrome therapy^53^.

Drugs toxicity in the CBM treatment and the increase of resistant strains have driven studies on a combined therapy^54^^,^^55^. The benefits of this therapy are well-known and include a broad spectrum efficacy, greater potency than monotherapy, reduction emergence of resistance as well as improvements in both safety and tolerability^56–58^. Considering ritonavir was the most effective in inhibiting peptidase activity, cellular growth and fungal viability after macrophage interaction, we also evaluated its action in combination with classical antifungal drugs against *P. verrucosa*. Under the conditions tested, a possible synergistic effect was observed since the association of ritonavir with ketoconazole and itraconazole promoted a greater inhibition in *P. verrucosa* growth. Our group previously demonstrated that the association between nelfinavir and amphotericin B highly inhibited *F. pedrosoi* development^13^. Our data also corroborate previous studies with other fungi. For instance, the combination of ritonavir or saquinavir with itraconazole showed a synergistic effect against *H. capsulatum*^59^. Also, saquinavir and fluconazole promoted a similar action against *C. albicans* and *C. neoformans*^60^. The association of PIs and antifungal drugs can potentially modify pharmacokinetic parameters due to the drug-drug interactions^61^. This may for instance increase drugs absorption, inhibiting enzymes that are responsible for their degradation, and consequently enabling high levels and prolonged action of drugs. Therefore, drugs with diverse mechanisms of action or therapies of multitarget combination should be objects of further studies to treat fungal infections^62^. In fact, peptidases are potential targets for antifungal drug development, since these enzymes play crucial metabolic and regulatory roles in several biological events of fungal cells^41^^,^^63^. PIs employment in fungal therapy is greatly encouraging, since they have already been used in clinical to treat hepatitis C and AIDS and had *in vitro* antifungal activity against opportunistic fungi^9^^,^^45^^,^^64^. Thus, drug repositioning emerges as an alternative therapeutic approach also to fungal infections^65^. However, it is important to point out that is relevant to develop more specific PIs for fungal cells, allowing their use at reduced dosage and toxicity. Thus, future studies will be focused on molecular docking experiments to predict the inhibitory potential and binding modes of HIV-PIs towards *P. verrucosa* aspartic peptidase. In addition, the purification of this fungal aspartic peptidase will be conducted to permit its crystallization and elucidation of three dimensional structure, which will contribute to synthesise more specific aspartic peptidase inhibitors. Taken together, our data showed that HIV-PIs were efficient in inhibiting important biological *P. verrucosa* processes and could be considered as a potential therapy, individually or in combination with classical antifungal agents, against neglected infections as those caused by this fungus.

## 5. Conclusion

Diseases caused by *P. verrucosa* can be chronic, recurrent and hard to treat. Studies have shown that several fungi secrete peptidases associated with crucial pathophysiological events. Thus, the detection of aspartic peptidase activity in *P. verrucosa* is important and the first step for the understanding of the possible role of this enzyme in metabolism- and infection-related processes. Our data clearly revealed that *P. verrucosa* had its growth especially inhibited by lopinavir and ritonavir, individually and in combination, even after macrophage interaction. In addition, our results corroborate other studies about the potential action of HIV-PIs as an alternative therapy for fungal infections.
